# Epidemiological trends and sociodemographic factors associated with acute hemorrhagic conjunctivitis in mainland China from 2004 to 2018

**DOI:** 10.1186/s12985-022-01758-6

**Published:** 2022-03-01

**Authors:** Rong Liu, Yuxing Chen, Hao Liu, Xihui Huang, Fang Zhou

**Affiliations:** 1 Institute for Disaster Management and Reconstruction, Sichuan University, Chengdu, 610000 China; 2 Institute of Chronic and Non-Communicable Disease Control and Prevention, Hubei Provincial Center for Disease Control and Prevention, No. 35 Zhuodaoquan North Road, Hongshan District, Wuhan, 430079 China; 3 Subject Teaching (English), College of Foreign Languages, Fujian Normal University, Fuzhou, 350000 China

**Keywords:** Acute hemorrhagic conjunctivitis, Trends, Disease hotspots, Sociodemographic factors

## Abstract

**Background:**

Acute hemorrhagic conjunctivitis (AHC) is classified as a class C notifiable infectious disease in China and poses a great threat to public health. This study aimed to investigate the epidemiological trends and hotspots of AHC in mainland China. Sociodemographic factors that could contribute to early warning of AHC were further explored.

**Methods:**

Yearly and monthly incidences of acute hemorrhagic conjunctivitis by date and region from 2004 to 2018 were extracted from the Data Center of China Public Health Science. Joinpoint regression and spatial autocorrelation analysis were performed to explore the epidemiological trends and hotspots of AHC. A generalized linear model was then applied to explore the relationship between sociodemographic factors and AHC incidence.

**Results:**

The average annual AHC incidence was 3.58/100,000 in mainland China. The first-level spatial and temporal aggregation areas were distributed in Guangxi, Hainan, Guangdong, Guizhou, Hunan, Jiangxi, Fujian, Chongqing, Hubei, Anhui, and Zhejiang, with gathering times from 2010/1/1 to 2010/12/31 (RR = 20.13, LLR = 474,522.89, P < 0.01). After 2010, the AHC incidence was stable (APC = − 8.37, 95% CI: − 23.02–9.06). However, it was significantly increased in low- and middle-income provinces (AAPC = 10.65, 95% CI: 0.62–21.68, AAPC = 11.94, 95% CI: 0.62–24.53). The peak of AHC occurred during the August to October period. Children who age 0–3 years are identified as high-risk group with AHC incidence significantly increased (APC = 31.54, 95% CI: 0.27–72.56). Birth rate, population ages 0–14 (% of total population), passenger traffic, and urban population (% of total population) were positively associated with the AHC incidence, while per capita gross domestic product was negatively associated with the AHC incidence.

**Conclusion:**

Overall, the AHC incidence was stable after 2010 in China, but it was significantly increased in low- and middle-income provinces. Regions with a high birth rate, population ages 0–14 (% of the total population), passenger traffic, urban population (% of the total population) and low per capita gross domestic product are at high risk of incidences of AHC. In the future, public health policy and resource priority for AHC in regions with these characteristics are necessary.

**Supplementary Information:**

The online version contains supplementary material available at 10.1186/s12985-022-01758-6.

## Introduction

Acute hemorrhagic conjunctivitis (AHC) is an acute viral eye disease that was first reported in Ghana in 1969 [1]. It is mainly caused by coxsackievirus A24 variant (CA24v) and human enterovirus type 70 (EV70), and it is characterized by bilateral eye pain, increased secretion, keratitis, and conjunctival congestion, commonly known as "pinkeye" [2]. Generally, the majority of cases present obvious symptoms but result in a favorable prognosis and rarely with severe complications or fatal infections [3–5]. Due to the rapid onset of symptoms, short incubation period and high infectivity, it usually leads to an outbreak or epidemic in the short term [6].

Since the first occurrence of AHC (caused by CA24v) in Singapore in 1970 [7], it has spread rapidly around the world. In recent decades, more than 10 million AHC cases have been reported worldwide [8], including several large outbreaks in Asia and South America. In 2002, the largest outbreak of AHC was reported in South Korea, with over 1,000,000 cases [9]. In 2014, an unprecedented nationwide outbreak of infectious conjunctivitis occurred in Thailand, which affected more than 300,000 individuals over 3 months [10]. In 2017, it was first reported in Mexico with 10,000 cases and then spread to other neighboring countries in the Caribbean and in South America, resulting in large outbreaks [2]. For instance, over 200,000 cases of AHC occurred in Brazil during the summer of 2017/2018 [13].

The economic and medical burden of conjunctivitis is also noteworthy. In France, during the 13-week epidemic, the estimated cost of the outbreak was €3,341,191 [14]. In the United States, 28% of annual emergency department visits are associated with conjunctivitis, amounting to approximately 560 million dollars in costs [15].

Although there has been no specific estimation of AHC costs in China to date, it still poses a great threat to public health. China has been classified as the most AHC-affected country worldwide [16]. Since the first cases were confirmed in 1971, many AHC cases have regularly appeared [17]. There have been 2 large outbreaks in different provinces. In 2007, the largest AHC epidemic in China was reported in Yunnan, which caused 74,263 cases [5]. Three years later, 69,635 cases caused by CA24v were reported in Guangdong [18]. To date, the AHC incidence ranked fifth among Class C notifiable diseases in 2020 [19].

Several studies have been performed to illustrate the epidemiological characteristics of AHC in China. However, most of the studies performed space–time analysis of AHC in a region-specific manner [20, 21]. Whether epidemiological trends of AHC change during long-term epidemics and what sociodemographic factors contribute to AHC has rarely been studied. Determining these questions is important because it helps to understand the dynamics of AHC and to determine where AHC is severe. Due to the lack of vaccines, identifying the sociodemographic factors associated with AHC could be integrated into the current surveillance system for early warning and risk prevention of AHC. To address these data gaps, we analyzed the long-term trends across time and space for AHC in mainland China using national notifiable infectious surveillance data from 2004 to 2018. We subsequently developed models to estimate the sociodemographic factors associated with AHC incidence.

## Materials and methods

### Data sources

#### Acute hemorrhagic conjunctivitis surveillance data

In this study, yearly and monthly AHC incidences (per 100,000) were applied for analysis, defined as the number of annual (monthly) incident cases divided by the population size [22]. The data were obtained by region from 2004 to 2018 from the Data Center of China Public Health Science, a publicly available web-based database established by the Chinese Center for Disease Control and Prevention (CDC) [23], which summarizes and sorts 31 provinces/municipalities/regions and national notifiable infectious surveillance data from the National Disease Supervision Information Management System (NDSIMS). This notifiable infectious disease routine reporting system was first established by the Chinese government in the 1950s and has been web-based since 2003, covering all populations in mainland China [22]. Physicians and local health authorities are required to register in this system within 24 h of diagnosis [24]. AHC incidence data retrieved from this system were also collected from all reported cases, and the definition of AHC was in accordance with the national diagnostic criteria based on the epidemiological history, clinical symptoms, and laboratory conjunctival cytology examination. (1) Epidemiological history: Most patients had a clear history of contact infection through eye-hand, object, and water-eye contact. Epidemiological history easily causes epidemics or outbreaks, commonly in the summer and autumn, and epidemic periods without seasonality. (2) Clinical symptoms, such as foreign body sensation in the eye, ocular redness, ocular stinging, photophobia, lacrimation and frequent discharge. (3) Laboratory examination: EV70 or CA24v viruses were isolated from conjunctival swabs or conjunctival scraping cultures and identified as EV70 or CA24v viruses by microneutralization tests. Indirect immunofluorescence techniques were used to detect EV70 or CA24v antigen in conjunctival smears or cell culture smears. Serum anti-ev70 or anti-ca24v antibody titers in recovered patients were more than 4 times higher than those in acute patients [2, 25].

#### The sociodemographic factors associated with acute hemorrhagic conjunctivitis

We aggregated the following list of sociodemographic factors from 31 provinces from the China National and Provincial Statistics Yearbook that were hypothesized to be associated with incidences of AHC, which has been demonstrated in previous studies [26–28]: birth rate, urban population (% of total population), population density, population ages 0–14 (% of total population); passenger traffic; per capita gross domestic product; and health workers (per 1000 people). These factors were chosen after a literature review of AHC and other infectious disease-related studies, also considering the data availability. Specifically, we hypothesized that population characteristics are an important factor affecting the spread of infectious disease; thus, we included birth rate, urban population (% of the total population) and population density in the model, which has been documented to be related to various infectious disease outbreaks [29–31]. In addition, based on previous AHC studies [18, 32, 33] and from the results of our joinpoint regression showing that young children are high-risk age groups, variable population ages 0–14 (% of the total population) was also included. Passenger traffic variable was included to represent transportation factors since modern transportation systems has been improving, promoting regional exchanges and mobility, which also promoting the spread of disease [34]. Per capita gross domestic product is one of the most common economic research factors that has been well documented in infectious or noninfectious diseases [31, 35], while AHC diagnosis, treatment, prevention, control and reporting are all directly based on the capacity of the health system [29, 34]. In this study, we used health workers (per 1000 people) to represent health system-related factors.

## Statistical analysis

### Spatial autocorrelation analysis

#### Retrospective spatiotemporal scan statistic

The retrospective spatiotemporal scan statistic [37] was applied to detect the space–time cluster of AHC based on the discrete Poisson model. Dynamic and two-dimensional spatiotemporal was established to scan the incidence of disease in 31 provinces/municipalities/regions. A movable cylindrical window was created in the map to scan cases, with the size and position of the window in dynamic change, and to calculate the difference in incidence between the area inside and outside the window. The H0 hypothesis was that disease is randomly distributed in space and time. The alternative hypothesis (H1 hypothesis) assumed that, compared to outside, the incidence of disease increased inside the scanning window. The differences in incidence between regions inside and outside the scanning window were calculated and tested by the log likelihood ratio (LLR) [38]. The scanning window with the largest LLR was identified as a high incidence cluster window. Study areas contained in the high incidence cluster window were found, and the relative risk (RR) was calculated. Monte Carlo simulation was used to assess whether LLR was significantly different from zero.

### Spatial autocorrelation analysis

Global and local spatial autocorrelation analyses were used to measure spatial autocorrelation [39]. This paper used global Moran’s I to detect the degree of spatial autocorrelation of AHC incidence and bivariate Moran's I to test the spatial autocorrelation between sociodemographic factors and AHC incidence from the whole region. In addition to above global Moran's I measurements, the Anselin local Moran’s I was also used to reflect the local spatial correlations within geographic units to consider the existence of spatial heterogeneity and thus to identify the hot/cold spots and outliers. Based on the fact that the spatial correlation may differ locally within a large study area [40].

For the results of local spatial autocorrelation analysis, Anselin local Moran’s I presented four categories of results: high-high cluster (high AHC incidence area surrounded by high AHC incidence area), low-low cluster (low AHC incidence area surrounded by low AHC incidence area), high-low cluster (high AHC incidence area surrounded by low AHC incidence area), and low–high cluster (low AHC incidence area surrounded by high AHC incidence area) [41]. The value of Moran's I ranges from − 1 to 1; specifically, if Moran's I > 0, there exists a positive spatial correlation; the larger this value, the stronger the correlation will be. Conversely, if Moran's I < 0, negative spatial correlation exists. The Z test and P values were used to assess significant differences.

### Joinpoint regression analysis

The joinpoint regression model [42] was used to examine the age distribution of AHC and its trends from 2004 to 2018 in mainland China. We further divided 31 provinces/municipalities/regions into 3 income groups (high-, middle-, low-income) by its ranks of per capita gross domestic product in 2018 [43]. Using the commonly used division method tertile to divide the ranks into three equal sections, the ranges 0–33.3%, 33.4–66.7%, 66.8–100% were defined as high-, middle- and low-income groups, respectively. The annual percentage change (APC) with its 95% confidence interval (CI) was calculated for each identified trend segment. The Z test was used to assess whether the APC was significantly different from zero. Significant APC used the terms “increase/decrease” to describe trends. Meanwhile, for nonsignificant APC, the term “stable” was used. We also estimated the average annual percentage change (AAPC) to describe long-term trends [29], assuming there was only 1 segment for the full range of our study period (2004–2018).

### Generalized linear model

The log-linear generalized linear model was used to fit the AHC incidence and 7 sociodemographic factor covariates during 2004–2018. The details are as follows:

$${\text{Log - linear}}:{\text{ lg(AHC}}\;{\text{incidence) }}\sim {\text{ norm}}({\text{lg}}\left( {\text{u}} \right),\sigma^{2} ),$$

where lg(u) = β_0_ + β_1*_ Year + β_2*_ Birth rate + β_3*_ (Population ages 0–14 (% of total population)) + β_4*_ Urban population (% of total population) + β_5*_ Population density + β_6*_ lg Passenger traffic + β_7*_ Per capita Gross domestic product + β_8*_ (Health workers (per 1000 people)), $\sigma$^2^ = variance of lg(AHC incidence).

The best-fitting model was determined according to the Akaike information criterion [44].

### Software

Microsoft Excel 2016 was used for data extraction, sorting, and cleaning, and SaTScan (version 9.5), Joinpoint (version 4.8.0.1), and R (version 3.6.1) were used for further data analysis. A brief outline of the study is shown in Additional file 1: Table S1.

## Results

### The incidence of acute hemorrhagic conjunctivitis in mainland China from 2004 to 2018

**Fig. 1:**
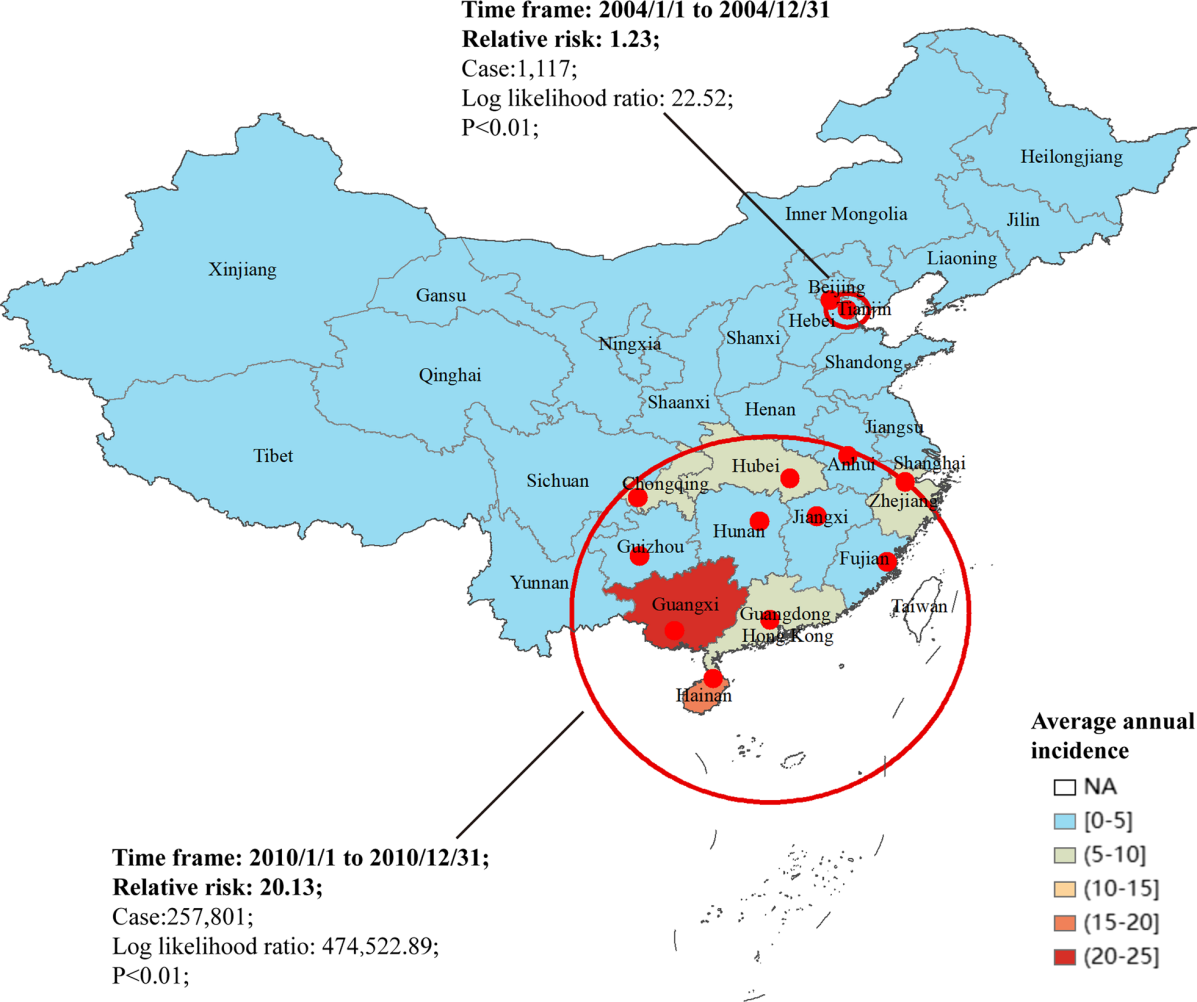
The incidence and space–time cluster of acute hemorrhagic conjunctivitis in mainland China, 2004–2018

In total, 720,640 cases were reported in mainland China from Jan 1, 2004 to Dec 31, 2018. The average annual incidence was 3.58/100,000. The top five incidence regions of AHC were Guangxi (20.91/100,000), Hainan (17.68/100,000), Guangdong (9.84/100,000), Chongqing (7.04/100,000), and Hubei (6.34/100,000) provinces (Fig. 1, Table 1).

**Table 1:** Incidence and trends of acute hemorrhagic conjunctivitis by 31 surveillance provinces/municipalities/regions in mainland China, 2004–2018

Region	Number of cases	Average annual incidence (/100,000)			AAPC	Trends
		2004–2018	2004–2009	2010–2018		
Guangxi	150,716	20.91	6.45	30.55	8.49 (− 3.45 to 21.91)	Stable
Hainan	23,477	17.68	4.00	26.8	31.80 (9.54–58.58)*	**Increased**
Guangdong	143,833	9.84	7.21	11.6	1.67 (− 12.17 to 17.69)	Stable
Chongqing	30,686	7.04	4.06	9.03	3.32 (− 6.58 to 14.27)	Stable
Hubei	54,810	6.34	2.03	9.22	19.95 (4.06 to 38.28)*	**Increased**
Zhejiang	49,228	6.32	4.55	7.50	− 4.47 (− 16.76 to 9.65)	Stable
Anhui	31,888	3.48	0.54	5.45	24.58 (12.48 to 37.98)*	**Increased**
Yunnan	23,438	3.36	1.85	4.37	26.12 (7.77 to 47.60)*	**Increased**
Sichuan	40,429	3.29	2.77	3.64	− 7.13 (− 17.59 to 4.67)	Stable
Hunan	29,099	2.97	1.26	4.11	10.69 (− 2.02 to 25.06)	Stable
Shaanxi	15,943	2.82	0.64	4.28	11.77 (− 8.72 to 36.87)	Stable
Guizhou	13,599	2.5	2.3	2.63	8.46 (0.56 to 16.99)*	**Increased**
Jiangxi	16,009	2.39	0.62	3.56	15.29 (2.78 to 29.32)*	**Increased**
Beijing	4882	1.99	4.07	0.61	− 24.17 (− 26.46 to − 21.81)*	*Decreased*
Fujian	10,815	1.96	0.69	2.81	8.04 (− 5.53 to 23.55)	Stable
Hebei	20,985	1.93	1.12	2.48	13.05 (10.02 to 16.16)*	**Increased**
Ningxia	1799	1.89	1.52	2.14	3.59 (− 0.98 to 8.38)	Stable
Henan	20,589	1.45	0.43	2.13	19.31 (12.96 to 26.02)*	**Increased**
Gansu	5087	1.31	0.35	1.94	22.75 (16.77 to 29.04)*	**Increased**
Qinghai	894	1.04	0.64	1.3	11.42 (7.48 to 15.51)*	**Increased**
Tibet	434	1.02	1.04	1.01	1.42 (− 19.07 to 27.10)	Stable
Shanghai	2396	0.86	1.12	0.69	− 22.84 (− 29.81 to − 15.17)*	*Decreased*
Jiangsu	9398	0.8	0.48	1.02	7.97 (− 6.48 to 24.65)	Stable
Shanxi	4066	0.76	0.48	0.95	3.75 (− 4.90 to 13.18)	Stable
Tianjin	1190	0.71	1.32	0.30	− 23.49 (− 26.5 to − 20.35)*	*Decreased*
Shandong	8695	0.6	0.2	0.87	18.59 (9.63 to 28.29)*	**Increased**
Inner Mongolia	1976	0.54	0.42	0.62	12.39 (2.3 to 23.47)*	**Increased**
Xinjiang	1111	0.36	0.64	0.17	− 15.15 (− 18.08 to − 12.12)*	*Decreased*
Jilin	1300	0.32	0.16	0.42	− 3.27 (− 20.67 to 17.94)	Stable
Liaoning	1264	0.19	0.14	0.23	4.92 (− 1.42 to 11.67)	Stable
Heilongjiang	604	0.11	0.07	0.13	0.15 (− 8.10 to 9.15)	Stable
Mainland China	720,640	3.58	1.82	4.75	8.04 (− 5.53 to 23.55)	Stable

*P < 0.05

### The space–time cluster of acute hemorrhagic conjunctivitis in mainland China from 2004 to 2018

The global and local spatial autocorrelation analysis results demonstrated a positive correlation of AHC incidence in mainland China. High-high aggregation areas were often found in South China (Additional file 1: Table S2 and Additional file 1: Fig. S1). Two spatial and temporal aggregation areas were revealed. The first-level spatial and temporal aggregation areas were distributed in Guangxi, Hainan, Guangdong, Chongqing, Hubei, Zhejiang, Guizhou, Hunan, Jiangxi, Fujian, and Anhui, with gathering times from 2010/1/1 to 2010/12/31 (RR = 20.13, LLR = 474,552.89, *P* < 0.01). The secondary spatial and temporal aggregation areas were Beijing and Tianjin from 2004/1/1 to 2004/12/31 (RR = 1.23, LLR = 22.52, *P* < 0.01) (Fig. 1).

### The incidence trends of acute hemorrhagic conjunctivitis in mainland China from 2004 to 2018

**Fig. 2:**
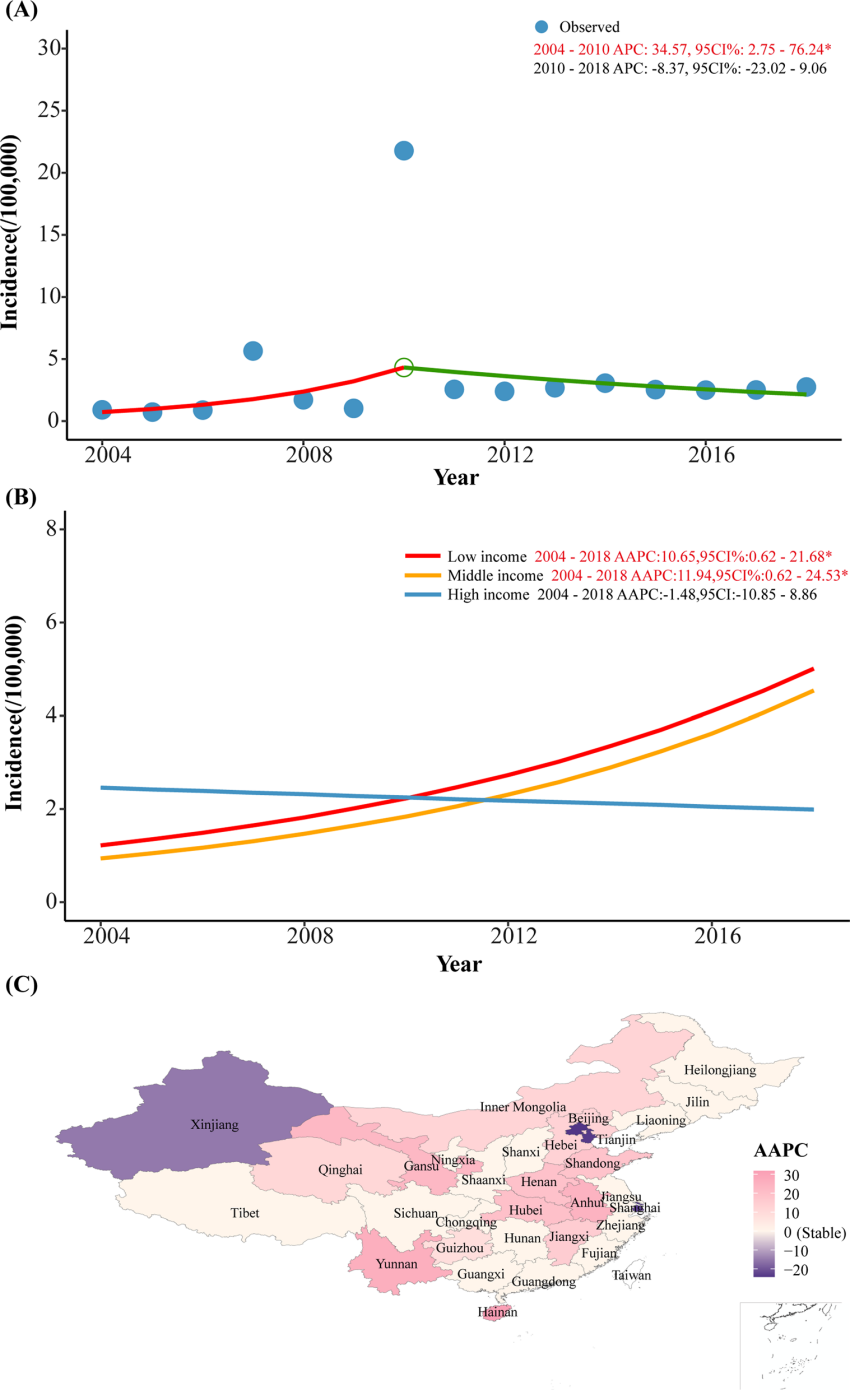
Trends in incidence of acute hemorrhagic conjunctivitis in mainland China, 2004–2018 (red font*: statistically significant trends; APC: annual percentage change; AAPC: average annual percentage change)

The joinpoint regression model revealed that the AHC incidence remained stable after 2010 (APC = − 8.37, 95% CI: − 23.02 to 9.06, *P* < 0.05). From 2004 to 2010, the incidence of AHC presented a significant increasing tendency, with an APC of 34.57 (95% CI: 2.75–76.24, *P* < 0.05) (Fig. 2a). From 2004 to 2018, 13 provinces showed significant increasing trends, including Hainan, Hubei, Yunnan, Anhui, Gansu, Hubei, Henan, Shandong, Jiangxi, Hebei, Inner Mongolia, Shaanxi, and Qinghai Guizhou, while 4 provinces showed significant decreasing trends, including Beijing, Tianjin, Shanghai and Xinjiang (Table 1, Fig. 2c).

When sub grouped by income level, the incidences of AHC in low and middle-income provinces were significantly increased (AAPC = 10.65, 95% CI 0.62–21.68; AAPC = 11.94, 95% CI 0.62–24.53, *P* < 0.05) (Fig. 2b).

### The seasonal and age group distribution of acute hemorrhagic conjunctivitis in mainland China from 2004 to 2018

**Fig. 3:**
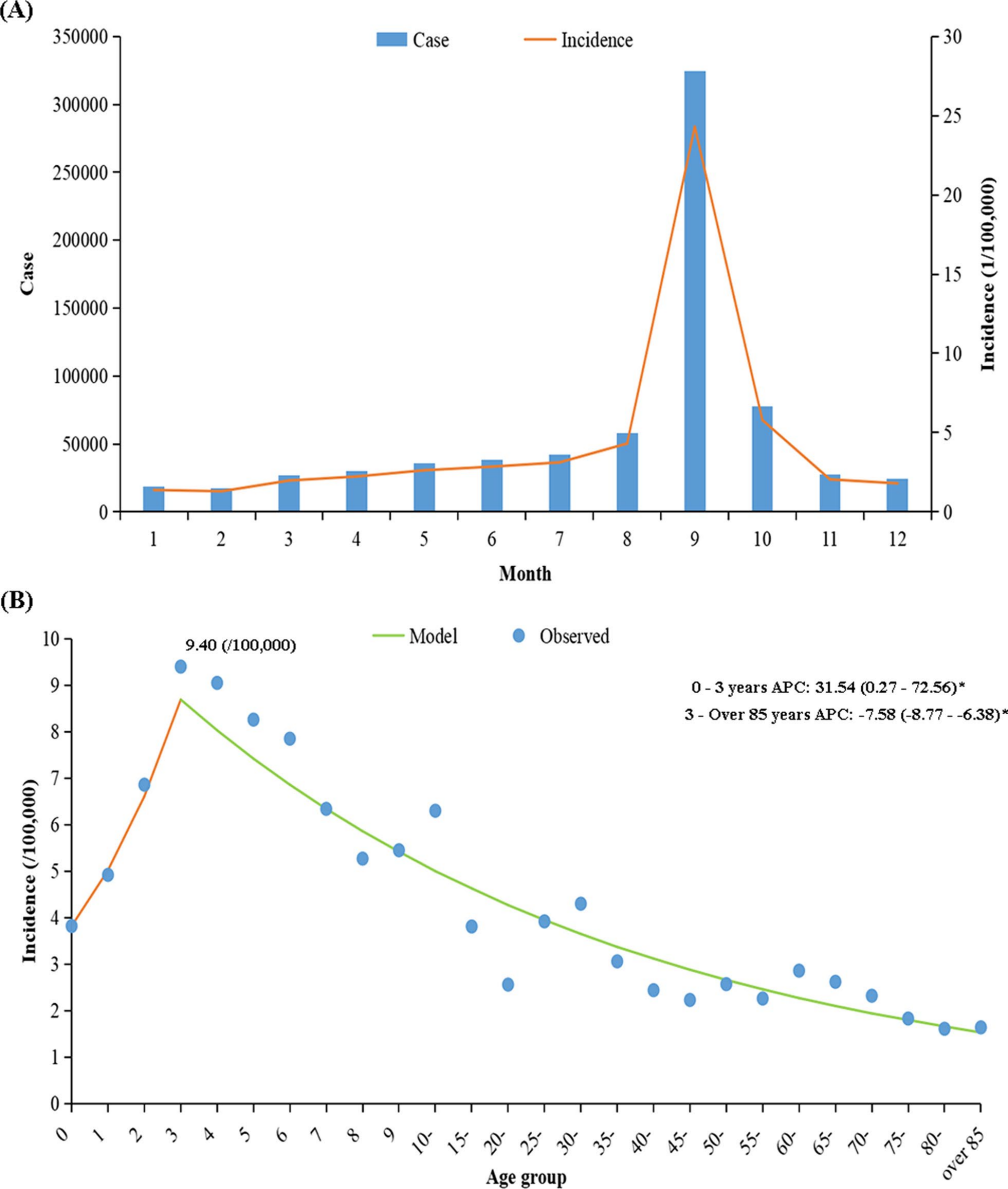
The seasonality and age group distribution of acute hemorrhagic conjunctivitis in mainland China, 2004–2018

We found a clear seasonality of AHC incidence, with one peak of AHC appearing from August to October (Fig. 3a). When examining age groups, children had a higher average annual incidence, especially in the age group of 3 years (9.40/100,000). AHC incidence was increased in the age of 0 to 3 (APC = 31.54, 95% CI: 0.27–72.56, *P* < 0.05), while after the age of 3, it was decreased (APC = − 7.58, 95% CI: − 8.77–6.38, *P* < 0.05) (Fig. 3b).

### Sociodemographic factors associated with the incidence of acute hemorrhagic conjunctivitis

Bivariate global spatial autocorrelation analysis and GLM both explored the association between sociodemographic factors and AHC incidence from different perspectives. The GLM results revealed that a high birth rate (*β* = 18.51, *P* < 0.001), population ages 0–14 (% of the total population) (*β* = 0.08, *P* < 0.05), urban population (% of the total population) (*β* = 0.08, *P* < 0.001), and passenger traffic (*β* = 0.12, *P* < 0.001) were positively associated with AHC incidence, while a low per capita gross domestic product (*β* = − 0.0004, *P* < 0.001) was negatively associated with AHC incidence (Table 2). The bivariate global spatial analysis results were mainly consistent with GLM results (Additional file 1: Table S3).

**Table 2:** The log− linear model for the association between sociodemographic factors and incidence of acute hemorrhagic conjunctivitis

Variables	Coefficient	Standard error	*P *value
Year	0.1654	0.0248	< 0.001**
Birth rate	18.513	4.5161	< 0.001**
Population ages 0–14 (% of total population)	0.0760	0.0376	0.044*
Urban population (% of total population)	0.0755	0.0119	< 0.001**
Population density	0.0001	0.0002	0.704
Log passenger traffic	0.1244	0.0267	< 0.001**
Gross domestic product per capital	− 0.0004	0.0000	< 0.001**
Health workers (per 1000 people)	− 0.0219	0.0594	0.106

**P < 0.001; *P < 0.05

## Discussion

Understanding the epidemiological distribution and specific spatiotemporal patterns of infectious diseases, also identifying its associated risk factors are the most important task for preventing and controlling infectious diseases [22]. In this study, we illustrate epidemiological trends, hotspots and sociodemographic factors associated with AHC in mainland China from an epidemiological and public health standpoint by using long-term population-based surveillance data. We reported that the epidemiological trends of AHC incidence significantly increased from 2004–2010 but stabilized after 2010. The incidence of AHC has its own characteristics. We found that the burden of AHC was relatively high in South China, particularly in Guangxi, Guangdong and Hainan provinces, and it was significantly increased in low- and middle-income provinces from 2004 to 2018. In addition, there was clear seasonality from August to October and a specific high-risk age group in children. Several sociodemographic factors were identified to be significantly associated with the AHC incidence. These findings will help government develop disease-specific and location-specific interventive measures in the following stage.

Zhang’s study reported that higher mean temperature, relative humidity and precipitation were associated with an increased risk of AHC [45]. Meteorological conditions in South China were also characterized by risk factors of high temperature, high relative humidity, and abundant rainfall, especially during summer, which were suitable for the growth and reproduction of intestinal viruses [46]. Therefore, in those high burden key provinces, strengthening epidemic surveillance by monitoring before the epidemic season, timely assessment and early warning of the epidemic development trend are critical for the prevention and control of AHC. This finding revealed that the AHC incidence increased from 2004–2010 but stabilized after 2010. Since 2004, China has established a notifiable infectious disease surveillance/reporting system with continuous standardization and maturity [22]. It has greatly promoted the prevention and control of 40 kinds of infectious diseases in China, including AHC. During the study period, there were two peaks of AHC incidence, in 2007 and 2010 [17]. Since 1971, Cox24v has been the major pathogen of AHC in China [47]. The outbreaks caused by CA24v are usually more widespread than those caused by EV70 [9]. From 1986 to 1988, CA24v caused epidemics in Beijing, Shanghai, and Guangzhou[48]. In 2007–2008, it was also responsible for outbreaks of AHC in Zhejiang, Yunnan and Guangdong [33, 49]. Two years later, the intensity of the outbreak in China caused by CA24v was significantly higher than that in 2007 and presented spatial–temporal clusters. G4-c3, G4-c5a and c5b of CA24v were the predominant strains in China from 2004 to 2014. They cocirculated and coevolved in southern and eastern China [16]. The genetic diversity of virus type alternation and antigen variation, lack of cross-protection between different genotypes, and poor immune persistence after infection may have resulted in random outbreaks of AHC [12]. In addition, the incidence of AHC was significantly increased in low- and middle-income provinces during the study period. Lower-income provinces are usually characterized by low immune systems, poor living environments, and poor knowledge of epidemic prevention measures, which might cause AHC to spread easily [50]. Regarding the AAPC, the provinces with higher increasing rates are also noticeable. Continuous surveillance to monitor genotypes and the emergence of new strains for the development of prevention and control strategies is necessary.

Children are usually the most frequently infected group [51]. In addition to school children, our results indicated that young children age 0–3 years are still worth studying. The absence of protective antibodies against enterovirus, low immunity and lack of basic hygiene knowledge in young children, along with poor knowledge of AHC among their parents (kindergarten teachers), leading to the inability to rapidly identify the main clinical symptoms, may result in higher risk [18, 52]. Therefore, health education should address hand-eye hygiene and improve the ability of parents (kindergarten teachers) to recognize the symptoms of conjunctivitis at an early stage.

Our results also provide insights into the relationship between sociodemographic factors and AHC, whose results emphasize the necessity of incorporating these parameters into the current surveillance system for early warning of AHC. Regions with high birth rates and populations aged 0–14 (% of the total population) contribute to high AHC incidence, echoing the results of high-risk age groups in the above discussion. China is rapidly becoming more urbanized, with low- and middle-income provinces experiencing the most striking social structure changes, which are also related to high AHC incidence, similar to other infectious diseases [31, 53, 54]. In addition to urbanization, passenger traffic greatly increases the risks of infectious disease spread, also including AHC [53]. Finally, yet importantly, regions with low per capita gross domestic products are at high risk of AHC, which may be characterized by poor sanitation permitting these causative agents to spread quickly [18, 50], echoing the results of the trends of AAPC subgroups by income. Those findings emphasize the necessity of incorporating these parameters into the current surveillance system for early warning of AHC. Characteristics related to the high incidence of AHC can also be applied to the prevention and control of AHC in other countries. Public health authorities should strengthen the surveillance of AHC in regions with these characteristics. Simultaneously, before the high-risk season, it is important to publicize and educate the public on the prevention and control of AHC, especially in high-risk groups. These efforts can help them understand the transmission and prevention methods of AHC, lend attention to personal eye hygiene, and form good hygiene habits and self-protection awareness. In addition, the infected should seek medical treatment as soon as possible and avoid going to public places, such school, nursery, factory, or natatorium [32].

## Limitation

This study has several limitations. First, in surveillance data, underreporting is a common problem. However, the dataset on the basis of the notifiable infectious disease reporting system used in this study was the most complete dataset currently available. Second, the sociodemographic factors identified in our study were only correlated with AHC, and a causal relationship was not proven. Further investigation is needed to determine the causality to provide a more precise basis when formulating targeted prevention policies. Last, this research only involved study site in China, more related-researches in different countries are also encouraged, then to provide more scientific evidence for prevent and control AHC, as well as other infectious diseases in future.

## Conclusion

AHC has a clear regional, population, and seasonal distribution in China during 2004–2018. The burden of AHC was high in South China, and children between the ages of 0–3 were at high risk, while the seasonal peak of AHC prevalence was from August to October. After 2010, the AHC incidence remained stable; however, in low- and middle-income provinces, the AHC incidence continuously increased. Regions with a high birth rate, population ages 0–14 (% of the total population), urban population (% of the total population), passenger traffic and low per capita gross domestic product are at high risk of AHC. In the future, public health policy and resource priority for AHC in regions with these characteristics are necessary.

## Supplementary Information

**Additional file 1: Table S1**. The brief outline of study. **Additional file 1: Table S2**. Global autocorrelation analysis of acute hemorrhagic conjunctivitis in mainland China, 2004–2018.**Additional file 1: Table S3**. Bivariate global Moran's I between sociodemographic factors and incidence of acute hemorrhagic conjunctivitis in mainland China, 2004–2018. **Additional file 1: Figure S1**. Spatial autocorrelation analysis of acute hemorrhagic conjunctivitis in mainland China, 2004–2018.

## Data Availability

The datasets used and/or analyzed during the current study are available from the corresponding author on reasonable request.

## Abbreviations

AHC: Acute hemorrhagic conjunctivitis
APC: Annual percentage change
AAPC: Average annual percentage change
RR: Relative risk
LLR: Log likelihood ratio
