# Supplementary material for: Epidemiological trends and sociodemographic factors associated with acute hemorrhagic conjunctivitis in mainland China from 2004 to 2018
Source: Virol J. 2022 Mar 1;19:34. doi: 10.1186/s12985-022-01758-6 (PMC8889670; doi:10.1186/s12985-022-01758-6)
Supplement: Supplementary file 1 — Additional file 1: Table S1. The brief outline of study. Additional file 1: Table S2. Global autocorrelation analysis of acute hemorrhagic conjunctivitis in mainland China, 2004–2018.Additional file 1: Table S3. Bivariate global Moran's I between sociodemographic factors and incidence of acute hemorrhagic conjunctivitis in mainland China, 2004–2018. Additional file 1: Figure S1. Spatial autocorrelation analysis of acute hemorrhagic conjunctivitis in mainland China, 2004–2018. [file 12985_2022_1758_MOESM1_ESM.doc]

Supplementary Table 1. The brief outline of study

| **Number** | **Objectives** | **Methods** | **Results** |
| --- | --- | --- | --- |
| 1 | To describe incidence of acute hemorrhagic conjunctivitis in mainland China from 2004 to 2018 | General descriptive analysis | Table 1 |
| 2 | To detect the space-time cluster of AHC incidence | Retrospective spatiotemporal scan statistic | Figure 1 |
| 2.1 | To detect the degree of spatial autocorrelation of AHC incidence from the whole region | Global spatial autocorrelation analysis | Supplementary Table 2 |
| 2.2 | To reflect the local spatial correlations by identity the hot/cold spots and outliers | Local spatial autocorrelation analysis | Supplementary Figure 1 |
| 3 | To explore the trends of incidence of acute hemorrhagic conjunctivitis from 2004 to 2018 | Join-point regression analysis | Figure 2 (A.B.C) |
| 4 | To revealed the seasonal and age distribution of acute hemorrhagic conjunctivitis from 2004 to 2018 | General descriptive analysis  Join-point regression analysis | Figure 3 (A. B) |
| 5 | To explore the association between sociodemographic factors and AHC incidence | Generalized linear model | Table 2 |
| 5.1 | To test the spatial autocorrelation between sociodemographic factors and AHC incidence from the whole region. | Bivariate global spatial autocorrelation analysis | Supplementary Table 3 |

**Supplemental Table 2. Global autocorrelation analysis of acute hemorrhagic conjunctivitis in mainland China, 2004 – 2018**

| **Year** | **Moran’s I** | **P** | **Z** | **Correlation** |
| --- | --- | --- | --- | --- |
| **2004** | 0.1070 | 0.1060 | 1.3105 | Not significant |
| **2005** | 0.113 | 0.096 | 1.2904 | Not significant |
| **2006** | 0.132 | 0.076 | 1.5931 | Not significant |
| **2007** | 0.190 | 0.018 | 2.4863 | Positive correlation |
| **2008** | -0.041 | 0.460 | -0.0536 | Not significant |
| **2009** | -0.014 | 0.3970 | 0.1823 | Not significant |
| **2010** | 0.157 | 0.029 | 2.3397 | Positive correlation |
| **2011** | 0.290 | 0.006 | 2.888 | Positive correlation |
| **2012** | 0.201 | 0.021 | 2.2756 | Positive correlation |
| **2013** | 0.197 | 0.020 | 2.3672 | Positive correlation |
| **2014** | 0.251 | 0.013 | 2.6973 | Positive correlation |
| **2015** | 0.234 | 0.016 | 2.5678 | Positive correlation |
| **2016** | 0.166 | 0.032 | 2.0679 | Positive correlation |
| **2017** | 0.204 | 0.015 | 2.3381 | Positive correlation |
| **2018** | 0.1620 | 0.024 | 2.2414 | Positive correlation |

**Supplementary Table 3.** **Bivariate global Moran's I between sociodemographic factors and incidence of acute hemorrhagic conjunctivitis** **in mainland China, 2004-2018.**

| **Year** | **Birth rate** | | | **Population ages 0-14**  **(% of total population)** | | | **Urban population**  **(% of total population)** | | | **Population density** | | |
| --- | --- | --- | --- | --- | --- | --- | --- | --- | --- | --- | --- | --- |
|  | Moran's I | Z-Score | P-Values | Moran's I | Z-Score | P-Values | Moran's I | Z-Score | P-Values | Moran's I | Z-Score | P-Values |
| 2004 | -0.0125 | -0.27 | 0.378 | -0.0579 | -0.7924 | 0.189 | 0.1133 | 1.4346 | 0.08 | 0.2384 | 2.7367 | 0.013 |
| 2005 | 0.0452 | 0.4389 | 0.344 | 0.001 | -0.0165 | 0.499 | 0.0658 | 0.8119 | 0.196 | 0.1985 | 2.1892 | 0.022 |
| 2006 | 0.0784 | 0.9323 | 0.175 | 0.0742 | 0.8677 | 0.19 | 0.0109 | 0.1791 | 0.397 | 0.1241 | 1.4313 | 0.076 |
| 2007 | 0.166 | 1.8022 | 0.036 | 0.2291 | 2.5675 | 0.009 | -0.1332 | -1.4374 | 0.07 | -0.0242 | -0.2307 | 0.429 |
| 2008 | 0.0824 | 0.9078 | 0.172 | 0.1054 | 1.2325 | 0.1 | -0.0102 | -0.0566 | 0.477 | 0.1090 | 1.2522 | 0.11 |
| 2009 | 0.0876 | 0.15 | 1.0477 | 0.1044 | 1.2689 | 0.103 | 0.0170 | 0.2510 | 0.393 | 0.1152 | 1.2764 | 0.101 |
| 2010 | 0.1660 | 1.954 | 0.023 | 0.2036 | 2.3503 | 0.011 | -0.0764 | -0.8302 | 0.205 | 0.0198 | 0.2022 | 0.388 |
| 2011 | 0.1381 | 1.629 | 0.053 | 0.1903 | 2.1722 | 0.02 | -0.0402 | -0.4357 | 0.321 | 0.0807 | 0.8869 | 0.189 |
| 2012 | 0.1410 | 1.6559 | 0.053 | 0.1772 | 2.0883 | 0.025 | -0.0789 | -0.8795 | 0.179 | -0.0121 | -0.1744 | 0.463 |
| 2013 | 0.1437 | 1.6963 | 0.051 | 0.1941 | 2.2397 | 0.018 | -0.0784 | -0.8807 | 0.179 | 0.0097 | 0.0688 | 0.421 |
| 2014 | 0.1658 | 1.9275 | 0.029 | 0.2663 | 3.0241 | 0.004 | -0.1717 | -1.9251 | 0.025 | -0.0376 | -0.4890 | 0.329 |
| 2015 | 0.2566 | 2.8214 | 0.004 | 0.2579 | 2.8610 | 0.004 | -0.1558 | -1.7498 | 0.041 | -0.0289 | -0.4010 | 0.36 |
| 2016 | 0.2125 | 2.3688 | 0.013 | 0.2263 | 2.5232 | 0.007 | -0.1113 | -1.2820 | 0.104 | -0.0083 | -0.1619 | 0.464 |
| 2017 | 0.2255 | 2.4774 | 0.01 | 0.2212 | 2.4533 | 0.011 | -0.1074 | -1.2361 | 0.108 | -0.008 | -0.1506 | 0.469 |
| 2018 | 0.2588 | 2.8011 | 0.002 | 0.2132 | 2.4248 | 0.008 | -0.1077 | -1.2499 | 0.106 | -0.0097 | -0.1795 | 0.466 |

Continue Table 2.

| **Year** | **Passenger traffic** | | | **Health workers (per 1,000 people)** | | | **Gross domestic product per capital (GDPP)** | | |
| --- | --- | --- | --- | --- | --- | --- | --- | --- | --- |
|  | Moran's I | Z-Score | P-Values | Moran's I | Z-Score | P-Values | Moran's I | Z-Score | P-Values |
| 2004 | 0.0075 | 0.1908 | 0.421 | 0.0569 | 0.9121 | 0.164 | 0.1637 | 1.9728 | 0.035 |
| 2005 | -0.0058 | -0.0286 | 0.498 | 0.0564 | 0.7776 | 0.2 | 0.13 | 1.5349 | 0.071 |
| 2006 | -0.0071 | -0.0796 | 0.486 | 0.0021 | 0.1138 | 0.408 | 0.0549 | 0.6894 | 0.226 |
| 2007 | 0.0587 | 0.8754 | 0.182 | -0.2047 | -2.2744 | 0.005 | -0.1575 | -1.7312 | 0.029 |
| 2008 | 0.0494 | 0.7246 | 0.195 | -0.1236 | -1.3527 | 0.051 | -0.0432 | -0.4238 | 0.37 |
| 2009 | 0.0414 | 0.6045 | 0.255 | -0.0718 | -0.7712 | 0.215 | -0.0067 | -0.0319 | 0.494 |
| 2010 | 0.1738 | 2.1721 | 0.034 | -0.1643 | -1.8398 | 0.009 | -0.1345 | -1.4849 | 0.049 |
| 2011 | 0.2685 | 3.2319 | 0.001 | -0.1809 | -2.007 | 0.019 | -0.1149 | -1.2658 | 0.106 |
| 2012 | 0.1989 | 2.3354 | 0.018 | -0.1626 | -1.8368 | 0.04 | -0.1596 | -1.768 | 0.036 |
| 2013 | 0.3028 | 3.5122 | 0.001 | -0.1452 | -1.6505 | 0.035 | -0.1513 | -1.6829 | 0.043 |
| 2014 | 0.2883 | 3.2583 | 0.002 | -0.1607 | -1.8387 | 0.032 | -0.1986 | -2.2123 | 0.014 |
| 2015 | 0.3102 | 3.5595 | 0.001 | -0.1260 | -1.4799 | 0.054 | -0.1677 | -1.8941 | 0.024 |
| 2016 | 0.3178 | 3.7052 | 0.001 | -0.0888 | -1.0723 | 0.126 | -0.12 | -1.4127 | 0.07 |
| 2017 | 0.3451 | 3.9998 | 0.001 | -0.0728 | -0.8916 | 0.166 | -0.0839 | -1.0402 | 0.143 |
| 2018 | 0.3119 | 0.001 | 3.6146 | -0.0287 | -0.4108 | 0.351 | -0.0751 | -0.9367 | 0.175 |


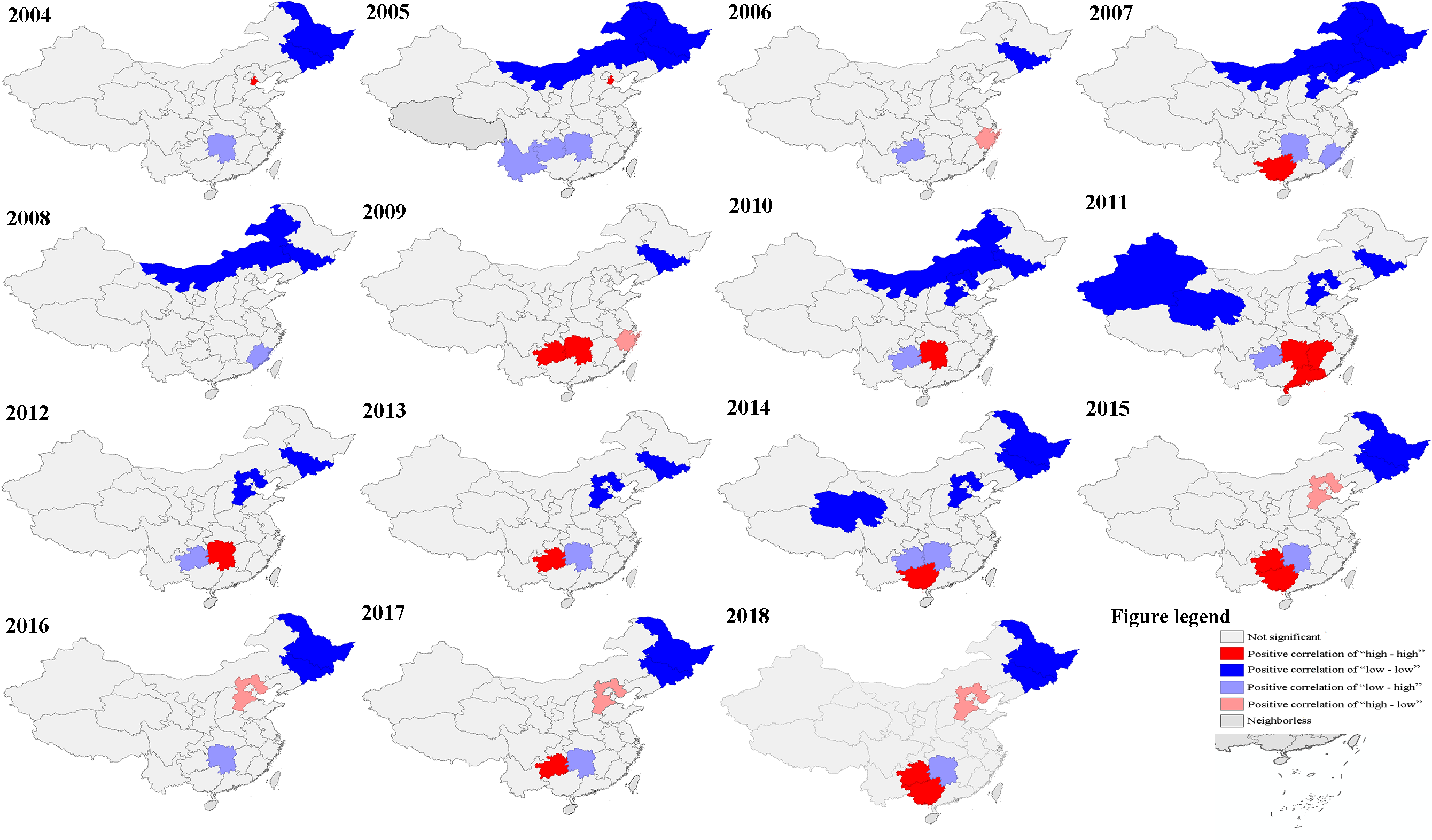


**Supplementary Figure 1. Spatial autocorrelation analysis of acute hemorrhagic conjunctivitis in mainland China, 2004 – 2018**
